# Patient-Reported Outcomes, Tumor Markers, and Survival Outcomes in Advanced GI Cancer

**DOI:** 10.1001/jamanetworkopen.2023.43512

**Published:** 2023-11-17

**Authors:** Joy X. Jarnagin, Anurag Saraf, Islam Baiev, Gary Chi, Emily E. van Seventer, Amirkasra Mojtahed, Jill N. Allen, Jeffrey W. Clark, Lawrence Blaszkowsky, Bruce J. Giantonio, Colin D. Weekes, Samuel J. Klempner, Joseph W. Franses, Eric J. Roeland, Lipika Goyal, Giulia Siravegna, Nora Horick, Ryan B. Corcoran, Ryan D. Nipp, Aparna R. Parikh

**Affiliations:** 1 Division of Hematology and Oncology, Department of Medicine, Massachusetts General Hospital Cancer Center and Harvard Medical School, Boston; 2 Department of Radiation Oncology, Massachusetts General Hospital and Harvard Medical School, Boston; 3 Department of Radiology, Massachusetts General Hospital and Harvard Medical School, Boston; 4 Department of Statistics, Massachusetts General Hospital and Harvard Medical School, Boston; 5 OU Health Stephenson Cancer Center, Section of Hematology and Oncology, Department of Internal Medicine, The University of Oklahoma (OU) College of Medicine, Oklahoma City

## Abstract

**Importance:**

Patient-reported outcomes (PROs), such as quality of life (QOL) and symptoms, are often associated with clinical outcomes in patients with cancer. In practice, oncologists use serum tumor markers (TMs) (ie, carcinoembryonic antigen [CEA] and carbohydrate antigen 19-9 [CA 19-9]) and imaging to monitor clinical outcomes in patients with gastrointestinal cancer.

**Objective:**

To examine associations of 1-month changes in PROs and TMs with treatment response and survival among patients with gastrointestinal cancer.

**Design, Setting, and Participants:**

This cohort study enrolled patients at Massachusetts General Hospital Cancer Center with at least 1 month follow-up from May 2019 to December 2020. Included patients were beginning first-line systemic therapy, aged 18 years or older, and had been diagnosed with metastatic pancreaticobiliary, colorectal, or gastroesophageal cancer. Data analyses took place from January 2021 to January 2022.

**Intervention:**

PROs were collected, including QOL (Functional Assessment of Cancer Therapy General [FACT-G]), physical symptoms (Edmonton Symptom Assessment System [ESAS]), and psychological symptoms (Patient Health Questionnaire-4 [PHQ4] total, PHQ4-depression, and PHQ4-anxiety), as well as TMs (CEA and CA 19-9), at the time of chemotherapy initiation and 1 month later.

**Main Outcomes and Measures:**

Associations of 1-month changes in PROs and TMs with treatment response (clinical benefit vs disease progression) at first scan, progression-free survival (PFS), and overall survival (OS), adjusted for baseline values using regression models.

**Results:**

This study included 159 patients, with 134 patients (84.3%) evaluable for analysis. Patients had a median (range) age of 64.0 (28.0-84.0) years and 86 (64.2%) were male. One-month PRO changes (FACT-G: OR, 1.07; 95% CI, 1.03-1.11; *P* = .001; ESAS-total: OR, 0.97; 95% CI, 0.94-1.00; *P* = .02; ESAS-physical: OR, 0.96; 95% CI, 0.92-1.00; *P* = .03; PHQ4-depression: OR, 0.67; 95% CI, 0.49-0.92; *P* = .01) were significantly associated with treatment response, but PHQ4-total or TMs were not. Changes in FACT-G (HR, 0.97; 95% CI, 0.95-0.99; *P* = .003), ESAS-total (HR, 1.03; 95% CI, 1.01-1.05; *P* = .004), ESAS-physical (HR, 1.03; 95% CI, 1.00-1.05; *P* = .02), PHQ4-depression (HR, 1.22; 95% CI, 1.01-1.48; *P* = .04), and CEA (HR, 1.00; 95% CI, 1.001-1.004; *P* = .001) were associated with PFS, but changes in PHQ4-total or TMs were not. Changes in ESAS-total (HR, 1.03, 95% CI, 1.01-1.06; *P* = .006) and ESAS-physical (HR, 1.04, 95% CI, 1.01-1.06; *P* = .015) were associated with OS, but changes in TMs were not associated with OS.

**Conclusions and Relevance:**

These findings suggest that 1-month changes in PROs can be associated with treatment response and survival in patients with advanced gastrointestinal cancer. Notably, 1-month changes in TMs were not consistently associated with these outcomes. These findings highlight the potential for monitoring early changes in PROs to associate with clinical outcomes while underscoring the need to address the QOL and symptom concerns of patients with advanced cancer.

## Key Points

**Question:**

Are early changes in patient-reported outcomes (PROs), such as quality of life and symptoms, as well as tumor markers, associated with clinical outcomes in patients with advanced gastrointestinal cancer?

**Findings:**

In this prospective cohort study of 159 patients with advanced gastrointestinal cancer, early changes in PROs (from baseline to 1 month) were associated with treatment response, progression-free survival, and overall survival, whereas tumor markers were not consistently associated with these outcomes.

**Meaning:**

These findings suggest that early changes in PROs may be associated with important outcomes among patients with advanced gastrointestinal cancer, underscoring the need to monitor and address quality of life and symptom concerns in this population.

## Teaser

This cohort study examines the association of 1-month changes in patient-reported outcomes and serum tumor markers with treatment response and survival among patients with gastrointestinal cancer.

## Introduction

Patient-reported outcomes (PROs) of quality of life (QOL) and symptoms are frequently associated with clinical outcomes for individuals with gastrointestinal cancers.^1,2^ Clinicians often use biomarkers, such as tumor markers (TMs) (ie, carcinoembryonic antigen [CEA] and carbohydrate antigen 19-9 [CA19-9]) and radiographic response, to guide clinical management,^3,4^ and novel biomarkers (ie, circulating tumor DNA [ctDNA]) are increasingly used. Although PROs are not typically conceptualized as biomarkers to inform clinical decision-making, they may represent a pragmatic strategy for assessing therapeutic benefits directly from the perspective of the patient.^1,5^ Furthermore, longitudinal changes in PROs may inform the need for proactive symptom management or alternative treatment strategies.^6^ However, a dearth of studies exist that investigate the association of changes in PROs with clinical outcomes in oncology.^6,7^

In this prospective cohort study, we investigated associations among 1-month changes in PROs (QOL and symptoms) and TMs with clinical outcomes (treatment response, progression-free survival [PFS], and overall survival [OS]) in individuals with advanced gastrointestinal cancers. We hypothesized that early changes in patients’ QOL, symptom burden, and TMs could associate with clinical outcomes, which may provide further support for the importance of routinely collecting and addressing longitudinal PROs in oncology clinical practice.

## Methods

This cohort study follows the Strengthening the Reporting of Observational Studies in Epidemiology (STROBE) reporting guideline. The Dana-Farber and Harvard Cancer Center institutional review board approved the study, and all participants signed informed consent.

### Participants and Study Procedures

We prospectively enrolled patients who were 18 years or older with metastatic pancreaticobiliary, colorectal, and gastroesophageal cancer with plans to initiate first-line systemic therapy at Massachusetts General Hospital (MGH) from May 2019 to December 2020 (eFigure in Supplement 1). We collected PROs and TMs at the time of chemotherapy initiation (within 7 days from treatment start) and 1 month (plus or minus 7 days) after starting treatment. Patients had to be able to read and respond to study questionnaires in English.

### Study Measures

#### Demographic and Clinical Information

We obtained sociodemographic data (eg, age, sex, race) and clinical information (eg, treatment details, tumor markers) from the electronic health record. Race and ethnicity were self-reported by the patient in the electronic medical record, and the categories included African American, Asian, Hispanic, White, and declines to provide. Although the study was not powered for race and ethnicity, we felt it was relevant data to collect for this study.

#### Patient-Reported Outcomes

We collected PROs assessing QOL (Functional Assessment of Cancer Therapy-General [FACT-G]),^8^ physical symptoms (Edmonton Symptom Assessment Scale-revised [ESAS-r],^9^ and psychological symptoms (Patient Health Questionnaire-4 [PHQ4]).^10^ We used the FACT-G to assess QOL (functional, physical, emotional, and social well-being), with scores ranging from 0 to 108 and higher scores indicating better QOL.^8^ FACT-G contains 27 items measuring the impact of disease on patient QOL across domains of functional, physical, emotional, and social well-being, such as questions regarding patient’s closeness to friends and family or patient’s worry about dying. For the ESAS-r, patients reported their symptoms on a Likert scale from 0 to 10, with 10 indicating the greatest severity. ESAS contains 11 items measuring the impact of disease on patient symptoms, including commonly affected symptoms, such as pain, tiredness, nausea, appetite, etc. We calculated a composite ESAS-total score (range of 0 to 110) and ESAS-physical score (range of 0 to 80) by summing all the symptom scores and the physical scores, with higher scores indicating worse symptoms. We added the symptoms of constipation and diarrhea to the ESAS-r, given the frequency of these symptoms in patients with gastrointestinal cancers.^11^ To evaluate depression and anxiety symptoms, we used the PHQ4 survey, with PHQ4-total scores ranging from 0 to 12 and higher scores indicating worse psychological distress.^10^ PHQ-total contains 4 items measuring the impact of disease on patient mood, specifically symptoms of anxiety and depression. We also used the PHQ4 to calculate subscores for depression (PHQ4-depression, consisting of the 2 questions regarding depression symptoms, scores ranging from 0 to 6) and anxiety (PHQ4-anxiety, consisting of the 2 questions regarding anxiety symptoms, scores ranging from 0 to 6) symptoms.

#### Treatment Outcomes

We classified treatment response on the first follow-up scan as either: (1) clinical benefit, defined as investigator-assessed tumor response or disease stability, or (2) disease progression, defined as increased tumor burden and/or clinical progression from baseline scan. We determined PFS using date of treatment initiation and dates of disease progression or death, with patients censored who were still on treatment at date of last follow-up (December 2, 2020). We estimated OS using date of treatment initiation and date of death and censored patients who were alive at the date of the last follow-up.

### Statistical Analysis

We calculated changes in PROs and TMs as the differences from baseline to 1 month after baseline. A negative value represented a value decreasing from baseline, and a positive value denoted a value increasing from baseline. We used logistic regression to examine associations among 1-month changes in PROs and TMs with the dichotomous treatment response outcome (clinical benefit or disease progression). We used Cox regression to investigate relationships among 1-month changes in PROs and TMs with survival outcomes (PFS and OS). We adjusted the baseline values of each respective PRO in the regression models. A 2-sided significance level of *P* < .05 was used for all comparisons, without adjustment for multiple comparisons given the exploratory nature of these analyses. The software package SAS version 9.4 (SAS Institute) was used to perform the statistical analyses. Analyses were conducted from January 2021 to January 2022.

## Results

### Participant Sample

During the study period, we enrolled 159 of 191 patients approached (83.2% enrollment), of whom 134 had baseline data and first follow-up data (PROs, TMs, and scan data). Participants had a median (range) age of 64.0 (28.0-84.0) years, with median (range) follow-up time of 13.5 (1.5-32.5) months. Of these 134 participants, 86 (64.2%) were male, 111 (82.8%) were White participants, 95 (70.9%) were married, and 82 (61.2%) were educated beyond high school (Table 1). The cancer subtypes were pancreaticobiliary (62 participants [46.3%]), colorectal (39 participants [29.1%]), and gastroesophageal (33 participants [24.6%]). The median (range) time to first scan was 2.01 (0.5-3.9) months (Table 1). Most patients had clinical benefit at the time of their first scan (85 participants [63.4%]). The median (range) PFS was 11.0 (1.0-189.0) months and median (range) OS was 13.5 (2.0-269.0) months.

**Table 1.** Patient, Disease, and Treatment Characteristics

Characteristic	Overall cohort (N = 134), No. (%)
Age, median (range), y	64.0 (28.0-84.0)
Sex	
Male	86 (64.2)
Female	48 (35.8)
Race	
Asian	9 (6.7)
African American	3 (2.2)
Hispanic	3 (2.2)
White	111 (82.8)
Declined to provide	8 (6.0)
Relationship status	
Married	95 (70.9)
Single	20 (14.9)
Divorced	9 (6.7)
Widowed	3 (2.2)
Other	4 (3.0)
Declined to provide	3 (2.2)
Education	
Beyond high school	82 (61.2)
High school and below	33 (24.6)
Declined to provide	19 (14.2)
Cancer type	
Pancreaticobiliary	62 (46.3)
Colorectal	39 (29.1)
Gastroesophageal	33 (24.6)
Treatment type	
Cytotoxic only	122 (91.0)
Targeted and cytotoxic	12 (9.0)
Current line of metastatic therapy	
1	98 (73.1)
2	18 (13.4)
≥3	18 (13.5)
Cancer response at first scan	
Clinical benefit	85 (63.4)
Disease progression	49 (36.6)
Months to scan (or clinical PD), mean (SD)^a^	2.01 (1.69)

^a^ Also includes days from treatment start to clinical disease progression if this occurred prior to the scan.

### One-Month Changes in PROs and Tumor Markers

The mean (SD) PHQ4-total scores (−0.6 [2.3]) decreased significantly from baseline to 1 month, whereas there was no change in mean (SD) FACT-G (−0.8 [10.7]), ESAS-total (−1.7 [15.3]), PHQ4-total (−0.6 [2.3]), PHQ4-depression (−0.08 [1.28]), and PHQ4-anxiety (−0.51 [1.46]), or ESAS-physical (0.03 [12.2]) scores from baseline to 1 month. Mean (SD) CEA (−2.2 [201.8]) and CA 19-9 (−6654.3 [6687.6]) scores also did not change significantly (Table 2).

**Table 2.** Changes in Patient-Reported Outcomes and Tumor Markers From Baseline to 1-Month After Initiation of Chemotherapy^a^

Variable	Within-patient change, mean (SD) [range]	95% CI
CEA	−2.2 (201.8) [−909.0 to 1840.5]	−38.40 to 33.93
CA 19-9	−6654.3 (66 876.1) [−618 340.0 to 58 878.0]	−20 823.95 to 7515.43
ESAS-total	−1.7 (15.3) [−61.0 to 37.0]	−4.47 to 1.14
ESAS-physical	0.03 (12.2) [−42.0 to 31.0]	−2.21 to 2.26
PHQ4-total	−0.6 (2.3) [−6.0 to 7.0]	−0.99 to −0.20
PHQ4-depression	−0.08 (1.28) [−4.00 to 3.00]	−0.30 to 0.15
PHQ4-anxiety	−0.51 (1.46) [−5.00 to 4.00]	−0.77 to 0.25
FACT-G	−0.8 (10.7) [−27.0 to 24.5]	−2.66 to 1.10

Abbreviations: CA 19-9, carbohydrate antigen 19-9; CEA, carcinoembryonic antigen; ESAS, Edmonton Symptom Assessment Scale; FACT-G, Functional Assessment of Cancer Therapy-General; PHQ4, Patient Health Questionnaire-4.

^a^ A negative change indicates a decrease in the value from baseline to 1 month, whereas a positive value indicates an increase from baseline to 1 month.

### Associations Among 1-Month Changes in PROs, Tumor Markers, and Treatment Response

Increases in FACT-G (OR, 1.07; 95% CI, 1.03-1.11; *P* = .001) and decreases in ESAS-total (OR, 0.97; 95% CI, 0.94-1.00; *P* = .02) and ESAS-physical (OR, 0.96; 95% CI, 0.92-1.00; *P* = .03) scores were associated with greater likelihood of clinical benefit at the time of first scans. Changes in PHQ4-depression scores (OR, 0.67; 95% CI, 0.49-0.92; *P* = .01) were significantly associated with clinical benefit, but changes in PHQ4-total (OR, 0.85; 95% CI, 0.72-1.01; *P* = .07) (Figure) and PHQ4-anxiety (OR, 0.91; 95% CI, 0.69-1.20; *P* = .50) scores were not. Changes in CEA (OR, 1.000; 95% CI, 0.998-1.002, *P* = .84) and CA19-9 (OR, 1.00, 95% CI, 1.00-1.00; *P* = .80) from baseline to 1-month were not significantly associated with treatment response.

**Figure.**
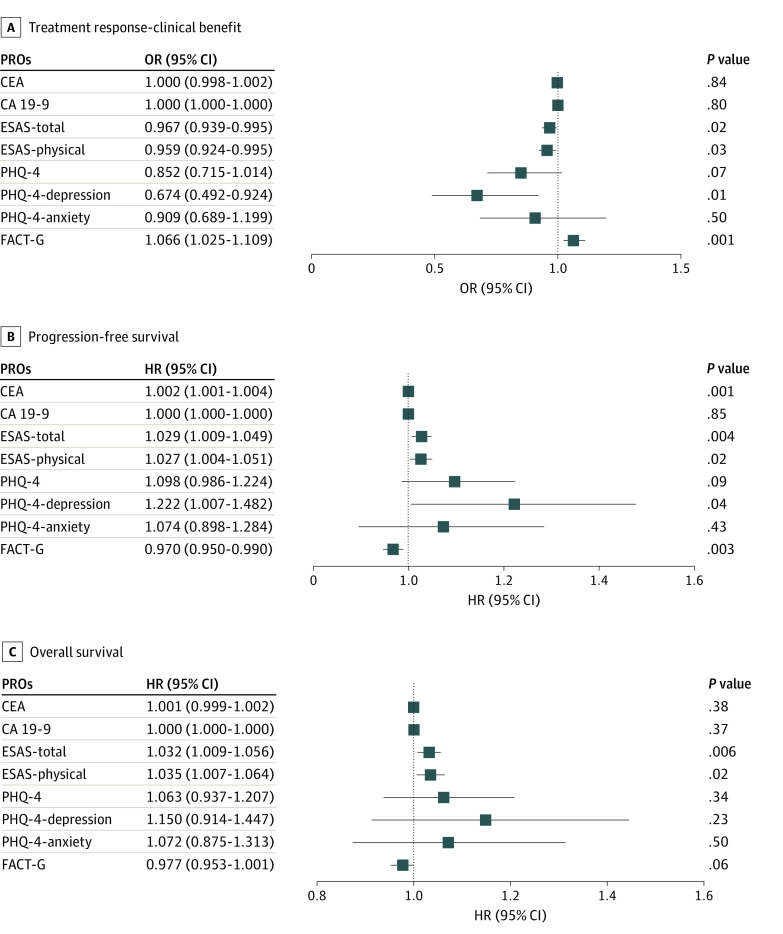
Association of Patient-Reported Outcomes (PROs) and Tumor Markers With Treatment Response, Progression-Free Survival, and Overall Survival CA 19-9 indicates carbohydrate antigen 19-9; CEA, carcinoembryonic antigen; ESAS, Edmonton Symptom Assessment Scale; FACT-G, Functional Assessment of Cancer Therapy-General; PHQ-4, Patient Health Questionnaire-4.

### Associations Among 1-Month Changes in PROs, Tumor Markers, and Survival Outcomes

Increases in FACT-G scores were associated with improved PFS (HR, 0.97; 95% CI, 0.95-0.99; *P* = .003) but not OS (HR, 0.98; 95% CI, 0.95-1.00; *P* = .06) (Figure). Increases from baseline to 1-month in ESAS-total (HR, 1.03; 95% CI, 1.01-1.05; *P* = .004) and ESAS-physical (HR, 1.03; 95% CI, 1.00-1.05; *P* = .02) were associated with worse PFS (Figure). Similarly, increases in ESAS-total (HR, 1.03; 95% CI, 1.01-1.06; *P* = .006) and ESAS-physical (HR, 1.04; 95% CI, 1.01-1.06; *P* = .02) were associated with worse OS (Figure). Changes in PHQ4-total scores were not significantly associated with PFS (HR, 1.10; 95% CI, 0.99-1.22; *P* = .09) or OS (HR, 1.06; 95% CI, 0.94-1.21; *P* = .34). Changes in PHQ4-depression scores were associated with PFS (HR, 1.22; 95% CI, 1.01-1.48; *P* = .04) but not OS (HR, 1.15; 95% CI, 0.91-1.45; *P* = .23). Changes in PHQ4-anxiety were not associated with PFS (HR, 1.07; 95% CI, 0.90-1.28; *P* = .43) or OS (HR, 1.07; 95% CI, 0.88-1.31; *P* = .50).

Increases in CEA from baseline to 1 month were associated with worse PFS (HR, 1.002; 95% CI, 1.001-1.004; *P* = .001), but not OS (HR, 1.001; 95% CI, 0.999-1.002; *P* = .38) (Figure). Changes from baseline to 1-month in CA19-9 were not significantly associated with PFS (HR, 1.000; 95% CI, 1.000-1.000; *P* = .85) or OS (HR, 1.000; 95% CI, 1.000-1.000; *P* = .38) (Figure).

## Discussion

In this prospective cohort study of patients receiving treatment for advanced gastrointestinal cancers, changes in PROs from baseline to 1 month after starting treatment were associated with several important clinical outcomes. Specifically, we found associations among changes in patient-reported QOL and symptoms with patients’ treatment response and survival outcomes. These findings suggest the association of early changes in PROs, while also highlighting the importance of addressing changes in patients’ symptoms and QOL when seeking to understand therapeutic benefits beyond radiographic assessments and tumor markers.

Our work underscores the importance of PROs in informing clinical management for patients with advanced cancer. Specifically, we found novel results suggesting that longitudinal changes in PROs can identify patients at higher risk for worse treatment response and survival outcomes. A more complete understanding of early changes in PROs could be instrumental in identifying patients who may benefit from proactive symptom management, early palliative care, and/or alternate treatment strategies to improve their outcomes.^1,12,13,14,15^ Our work supports the need for efforts to integrate early monitoring and management of PROs in patients with advanced gastrointestinal cancer.

To our knowledge, the current study is one of the first to report that early longitudinal changes in PROs are associated with treatment response and survival outcomes in patients with advanced cancer. Prior research has highlighted that PROs at a single time point are associated with tolerance of treatment and survival in patients with cancer.^2,12,13,16,17^ Additionally, previous work suggests that patients with cancer experience worsening of symptoms, such as anxiety, depression, fatigue, pain, and physical function, as they approach the end of life.^6^ In our current study, early changes in PROs (from baseline to 1 month) are associated with overall survival, clinical response, and progression-free survival, suggesting PROs as a potential biomarker for cancer-specific survival outcomes.

Importantly, the current study found associations of several distinct PROs with treatment response and survival outcomes, while showing the inconsistent ability for tumor markers to associate with these outcomes. We found that CEA was associated with PFS but not clinical benefit or OS, while CA 19-9 was not associated with any of these clinical endpoints. TMs represent an imprecise biomarker in clinical practice because not all patients have elevated TMs at diagnosis, and physiological conditions may impact the detection and clearance of TMs.^16,17^ Furthermore, TMs can exhibit a delay of 1 month or more in response to treatment, and longer duration of change in TMs may be aligned with clinical outcomes.

Of note, not all PROs were consistently associated with treatment response and survival outcomes. For example, changes in QOL scores were associated with clinical benefit and PFS but not OS, and changes in PHQ4-total scores were not associated with clinical benefit, PFS, or OS. However, ESAS symptom burden scores were associated with treatment response and survival outcomes. These findings suggest that early changes in ESAS scores may be more associated with cancer treatment response than other PROs. Additionally, we previously found that baseline PHQ4-total scores were associated with clinical outcomes,^2^ and our current findings suggest that early changes in PHQ4-total scores were less associated with clinical outcomes.^18,19,20^ Thus more research is needed to fully understand the potential underlying mechanism.

Within PHQ4 subscores, PHQ4-depression scores were associated with treatment response and PFS and were not associated with OS, while PHQ4-anxiety scores were not associated with treatment response, PFS, or OS. Research investigating changes in depression and anxiety symptoms in patients with cancer is limited, and further research should investigate changes in depression and clinical outcomes. Our findings suggest additional research among other cancer types and across different sites is warranted. Early changes in PROs were assessed in this study, and future work should analyze longitudinal data to understand the role of PROs and TMs in patients with gastrointestinal malignant neoplasms.

### Limitations

This study has limitations. The single institution design and lack of sociodemographic diversity limits the generalizability of our results. This study only included patients with 3 gastrointestinal diseases, 2 of which (pancreas and gastroesophageal) often have advanced symptoms when metastatic; thus 1-month changes may be amplified and limit the applicability to other cancers. Additionally, the limited sample size restricted our power to detect statistical significance and conduct robust subgroup analyses. Furthermore, PROs, such as coping, self-efficacy, and health literacy, may also influence patients’ clinical outcomes and were not assessed in this study.^21,22,23,24^

## Conclusion

In this cohort study, early changes in PROs were associated with treatment response and survival outcomes in patients with advanced gastrointestinal cancer. This work adds to the growing body of evidence supporting the routine implementation of PROs into oncologic care. These findings suggest the potential value of early changes in PROs and clinical outcomes among patients with advanced cancer, while also underscoring the importance of monitoring QOL and symptom concerns.

## Notes

Supplement 1. **eFigure.** CONSORT Diagram

Supplement 2.
Data Sharing Statement
