# Supplementary material for: Patient-Reported Outcomes, Tumor Markers, and Survival Outcomes in Advanced GI Cancer
Source: JAMA Netw Open. 2023 Nov 17;6(11):e2343512. doi: 10.1001/jamanetworkopen.2023.43512 (PMC10656643; doi:10.1001/jamanetworkopen.2023.43512)
Supplement: Supplement 1. — eFigure. CONSORT Diagram [file jamanetwopen-e2343512-s001.pdf]

Supplemental Online Content

Jarnagin JX, Saraf A, Baiev I, et al. Patient-reported outcomes, tumor markers, and survival outcomes in advanced GI cancer. *JAMA Netw Open*. 2023;6(11):e2343512
doi:10.1001/jamanetworkopen.2023.43512

eFigure. CONSORT Diagram

This supplemental material has been provided by the authors to give readers additional information about their work.

© 2023 Jarnagin JX et al. *JAMA Network Open.*

Supplemental Figure 1. CONSORT Diagram

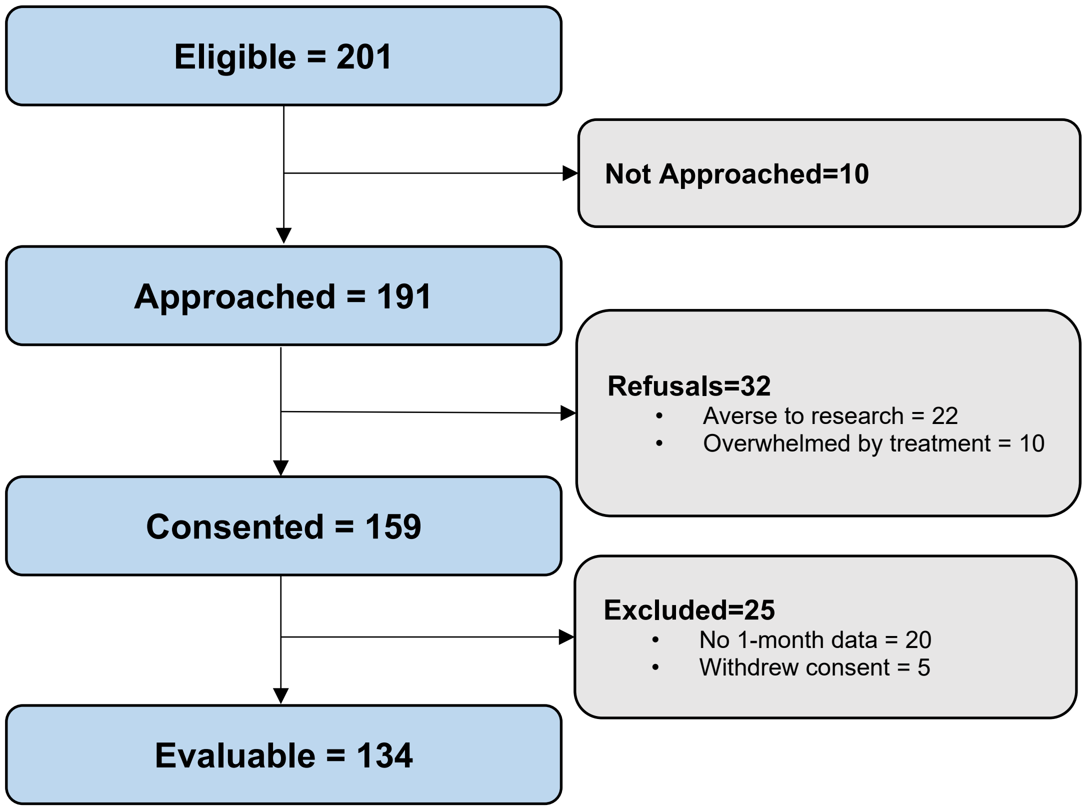

© 2023 Jarnagin JX et al. *JAMA Network Open*.
