# Supplementary material for: Patient-Reported Outcomes, Tumor Markers, and Survival Outcomes in Advanced GI Cancer
Source: JAMA Netw Open. 2023 Nov 17;6(11):e2343512. doi: 10.1001/jamanetworkopen.2023.43512 (PMC10656643; doi:10.1001/jamanetworkopen.2023.43512)
Supplement: Supplement 2. — Data Sharing Statement [file jamanetwopen-e2343512-s002.pdf]

Data Sharing Statement

Jarnagin. Patient-Reported Outcomes, Tumor Markers, and Survival Outcomes in Advanced GI Cancer. *JAMA Netw Open*. Published November 17, 2023. doi:10.1001/jamanetworkopen.2023.43512

Data
Data available: Yes
Data types: Deidentified participant data, Data dictionary
How to access data: Aparna.Parikh@mgh.harvard.edu
When available: With publication

Supporting Documents
Document types: None

Additional Information
Who can access the data: anyone requesting the data
Types of analyses: for a specified purpose
Mechanisms of data availability: after approval of a proposal and a signed data access agreement
